# Effect of technological parameters on mechanical properties and microstructure of heat-assisted friction stir welded joints of 6061 aluminum alloy

**DOI:** 10.1371/journal.pone.0334979

**Published:** 2025-10-22

**Authors:** Hoang-Linh Nguyen, Van-Trung Pham, Duc-Binh Luu, Thien-Phuc Tran

**Affiliations:** 1 Faculty of Mechanical Engineering, The University of Danang, University of Science and Technology, Danang City, Vietnam; 2 Department of Mechanical Engineering, Pham Van Dong University, Quang Ngai Province, Vietnam; 3 Faculty of Mechanical Engineering, Ho Chi Minh City University of Technology (HCMUT), Ho Chi Minh City, Vietnam; 4 Vietnam National University Ho Chi Minh City, Linh Trung Ward, Thu Duc City, Ho Chi Minh City, Vietnam; Institute of Infrastructure Technology Research and Management, INDIA

## Abstract

This study investigates the influence of key technological parameters on the mechanical characteristics and microstructure of heat-assisted friction stir welded (FSW) joints of AA6061 aluminum alloy pipes. Specifically, the effect of tool rotation speed, transverse speed, and tool shoulder diameter was evaluated. An experimental campaign was conducted using a three-level, three-factor composite design, with the primary objective of determining the optimal combination of these parameters to maximize the tensile strength of the weldments. AA6061 aluminum alloy pipes, with a thickness of 5 mm and an outer diameter of 80 mm, were joined using a resistance preheating FSW (RPFSW) process. The input parameters were varied at three distinct levels: rotation speed (1250, 1500, 1750 rpm), transverse speed (75, 87.5, 100 mm/min), and shoulder diameter (12, 15, 18 mm). Tensile tests were conducted to evaluate mechanical strength, and optical microscopy together with scanning electron microscopy (SEM), was employed to examine the microstructure of the weldments. The results indicate that the weldments achieved a tensile strength ranging from 49.7% to 72.4% of the base material. The optimal processing parameters were identified to achieve the highest predicted tensile strength of 198.15 MPa, corresponding to a transverse speed of 100 mm/min, a rotation speed of 1629 rpm, and a shoulder diameter of 13 mm. Microstructural analysis revealed that appropriate RPFSW parameters lead to suitable temperature and material flow, which in turn reduces weld defects and enhances the overall mechanical properties of the joint.

## Introduction

Aluminum and aluminum alloys are popular metals and are used in aerospace, automobile industry, railways, shipbuilding, electricity, construction, and so on [1]. AA6061 aluminum alloy is quite commonly used in the aerospace industry, significantly reducing weight while still ensuring high durability and saving a lot of fuel when operating [2]. Using aluminum alloys for welding by fusion welding methods is very difficult; for example, plasma arc welding has a defect rate of up to 90%. In 1991, the Welding Institute of the United Kingdom invented a new welding technology: friction stir welding (FSW) [3]. This solid-state (non-melting) welding process uses frictional heat, mainly applied to nonferrous metal materials, especially aluminum alloys [4]. FSW offers many benefits compared to traditional welding methods, such as electric welding, gas welding, or arc welding, including energy savings and environmental protection without creating arcs and welding fumes..., does not consume welding gas, does not need to use welding rod metal to fill the weld, minimizes deformation, does not crack, and ensures weld quality [5]. The friction stir welding has reduced this defect rate to almost zero [6].

It has been demonstrated that FSW is an effective joining technique for nearly all aluminum alloys. In addition to combining similar alloys of Al, FSW has been shown to be incredibly effective when joining different alloys of Al [7,8]. Furthermore, the FSW process has also been shown to be an effective joining method for many other alloys, such as those of magnesium and copper, and between different alloys [9–11]. Many researchers have recently focused on friction stir welding with nonferrous metals and metals of different compositions. Such as Jayakumar et al. [12] investigated the metallurgical, mechanical, and corrosion properties of dissimilar friction stir welded AA 6061-T6 and AA 5052-H32 aluminum alloys. According to the study, the joint formed at 950 rpm produced an ultimate strength of 174.3 MPa, a greater hardness of 72 HV compared to other joints, and a percentage elongation of 13.8%. In addition, Jayakumar et al. investigated the impact of some other parameters, such as tool pin and thickness of scandium intermetallic layer in friction stir welds [13–16].

Previous studies have demonstrated that the welding tool rotation speed, transverse speed, tool shoulder diameter, welding pressure, temperature, and the type of base material significantly influence the quality of FSW joints. For instance, Bandhu et al. [17,18] applied the Taguchi approach to investigate the impact of these parameters on the joint strength of high-density polyethylene sheets. Their findings indicated that a tool traverse speed of 20 mm/min, a rotation speed of 1070 rpm, and a tool tilt angle of 2° constituted the optimal combination of process parameters. More recently, Fuse et al. [19] utilized machine learning methodologies—including Adaptive Boosting, Decision Tree, k-Nearest Neighbors, Random Forest, Support Vector Machines, and Gaussian Naïve Bayes—to predict the tensile strength of aluminium alloys in FSW. Their findings highlighted that critical input variables such as axial force, tool shoulder diameter, tool rotation speed, traverse speed, plate thickness, and pin diameter strongly influence the mechanical performance of the weld. In parallel, the application of FSW to polymeric and composite materials has increasingly attracted attention due to its potential to overcome the limitations of conventional joining methods. Kurami et al. [20] provided a comprehensive review summarizing tool configurations and microstructural analysis techniques for these materials, thereby emphasizing the emerging opportunities and challenges in extending FSW beyond metallic alloys.

Applying FSW on aluminum alloys poses a big challenge to the problem of welding tool wear [21]. This problem can be solved by incorporating an external heat source to the FSW to improve the materials’ thermoplasticization process. Laser-heated FSW has been found to have many advantages, such as improved microstructure and a 10% improvement in tensile strength. The laser can raise the temperature of the stirring zone, reduce tool wear, and reduce the loading force of tools, but the major disadvantage is that the cost of laboratory equipment is too high [22–24]. Yaduwanshi et al.’s study [25] examined how preheating affects the qualities of the weld joint while joining aluminum alloy using the plasma-assisted FSW procedure. It is discovered that the preheating action improves material flow, and the plasma arc’s dissolving of small oxide particles causes an increase in strength and an alteration of the deformation behavior. Preheating increases the average hardness value and distributes hardness equally throughout the weld zone. Cota et al. [26] investigated the material flow characteristics of 5052 H34 aluminum alloy and SAE 1020 steel during the hybrid FSW method with plasma assistance. Compared to friction welding without heating, the results show that friction welding with heating tends to decrease the loading force and increase the temperature outside the stirring zone. Although plasma-heated FSW provides better mechanical weldment properties, controlling the temperature of plasma heating is difficult. The heating temperature from plasma is very high, so aluminum material quickly reaches its melting point. Naji et al. [27] studied the ultrasonic heat-assisted friction stir welding. Ultrasonic heat-assisted FSW offers many advantages over conventional FSW. High vibration frequencies improve the weldment quality, tribological, and mechanical properties of the weldment. It has been determined that FSW-assisted ultrasonic preheating significantly improves mechanical properties and material flow in the weld zone, reducing or eliminating some weld defects and enhancing joint mechanical properties and microstructure. Besides aluminum welding, ultrasonic heat-assisted FSW can be successfully implemented between hard metals such as titanium and steel. The disadvantage of the method is that the equipment cost is relatively high, and it is only suitable for welding thin materials.

Besides the aforementioned preheated friction stir welding techniques, resistance preheating friction stir welding (RPFSW) has demonstrated numerous advantages. Resistance preheating helps improve control over the workpiece’s temperature distribution. It helps in reducing residual stresses and distortion, resulting in higher-quality welds with improved mechanical properties. Because of their high melting points or heat conductivity, some materials—like titanium alloys, high-strength steels, and aluminum alloys—are difficult to weld with FSW. Resistance preheating helps soften these materials, making them easier to weld and reducing the wear and tear on the welding tool. Moreover, resistance heating systems can be integrated with advanced control systems to regulate the preheating temperature precisely. This precise control helps maintain consistent welding conditions, leading to more uniform and reliable welds. Compared to other preheating methods, resistance heating equipment can be less expensive and easier to maintain. This makes it an attractive option for industries looking to implement FSW without substantial additional investment in heating technology. Kaushik Sengupta et al. [28] have studied the FSW process with resistance preheating. The article presents the effects of welding parameters on weld quality, such as welding tool geometry, tool material, and welding process parameters. It has been demonstrated that FSW with resistance preheating of high-strength alloys, such as aluminum is an emerging technology with many commercial applications. Research also shows many advantages of this method compared to other heat-assisted FSW methods, such as increased tool life and performance, low energy consumption, low cost, ease of use, no loss of alloying elements during the joining process. Therefore, RPFSW research aimed at overcoming the disadvantages encountered by other heat-assisted FSW methods is appropriate and necessary.

AA6061 aluminum pipes are extensively employed in various engineering applications, including exhaust and air duct systems in the automotive industry, fuel and lubricating oil pipelines in the aerospace sector, and exhaust and ventilation pipes in the marine industry. Several studies on the FSW of aluminum alloy pipes have been reported previously [29–32]. However, most of these investigations kept all process parameters constant while varying only a single parameter at a time during optimization. These optimization methods and practices were not only time-intensive but also unsuitable for addressing the conflicting nature of multiple process parameters. Furthermore, although preheated friction stir welding offers many advantages in terms of improving material flow, reducing defects, and enhancing joint performance, there is a paucity of research on the parametric optimization of preheated FSW for aluminum pipes. Therefore, in this study, the Box–Behnken design is employed to optimize critical process parameters, namely transverse speed (mm/min), rotation speed (rpm), and tool shoulder diameter (mm), in the RPFSW of AA6061 aluminum alloy pipes. With the results achieved in the article, it is hoped that this weld can replace the riveted joints commonly used in the manufacture of aircraft and high-speed trains when using aluminum alloys.

### Experiments

#### Base material and specimens.

**Fig 1:**
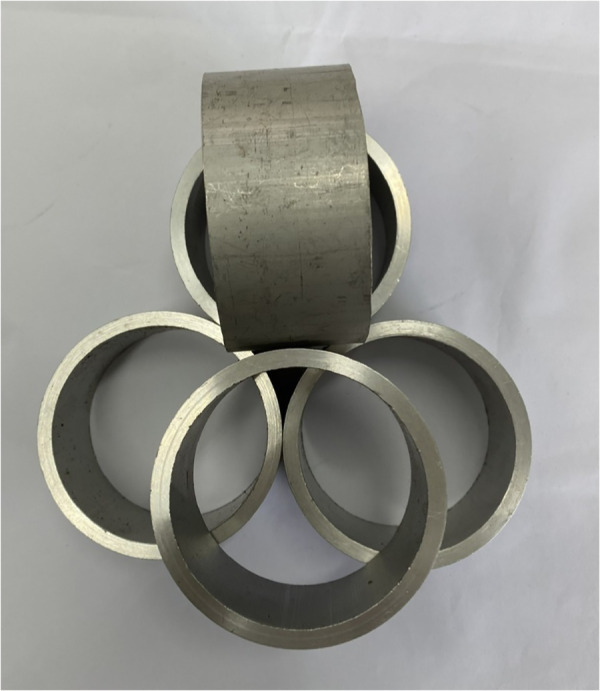
Specimens.

The weld workpiece material is AA6061 aluminum alloy pipes with dimensions: width of 40 mm, outer diameter of 80 mm, and thickness of 5 mm, as presented in Fig 1. Specimens were cut in a lathe machine from a 2.5 m long tube to ensure parallelism on both sides when mounted on the mandrel.

Before performing the experiment, we rechecked the chemical composition of the material. Table 1 displays the chemical composition of the aluminum alloy base material. The chemical composition is quite similar to previous studies [33–35].

**Table 1:** Chemical composition of AA6061 material.

Element	Al	Zn	Mg	Cu	Si	Fe	Mn	Cr	Ti	Others
Composition (%)	97.3	0.32	0.71	0.16	0.6	0.48	0.23	0.04	0.02	0.14

The above aluminum alloy base material has been tested for mechanical properties, as shown in Table 2. This result is consistent with previous studies [35,36]

**Table 2:** Tensile characteristics of AA6061 material.

Yield strength (MPa)	Ultimate strength (MPa)	Elongation (%)
230.2	273.8	12

**Fig 2:**
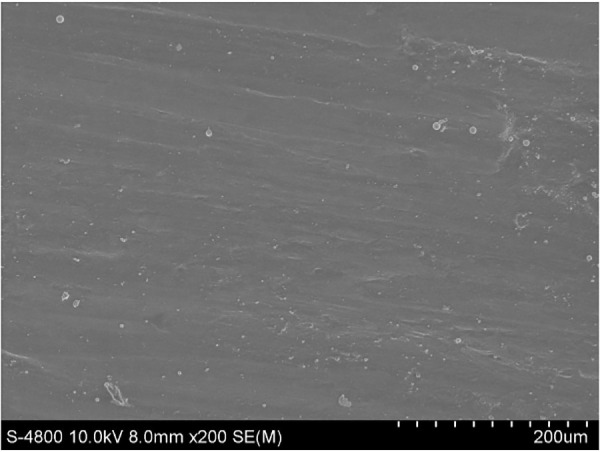
SEM image of the parent metal (AA6061).

The provided Scanning Electron Microscopy (SEM) image of AA6061 aluminum alloy reveals the material’s microstructure characteristics, as shown in Fig 2. There are no significant variations or abnormalities in the grain structure, suggesting a homogeneous processing condition. The uniformity in grain structure suggests controlled processing conditions, ensuring consistent mechanical properties across the material.

### Welding tools

**Fig 3:**
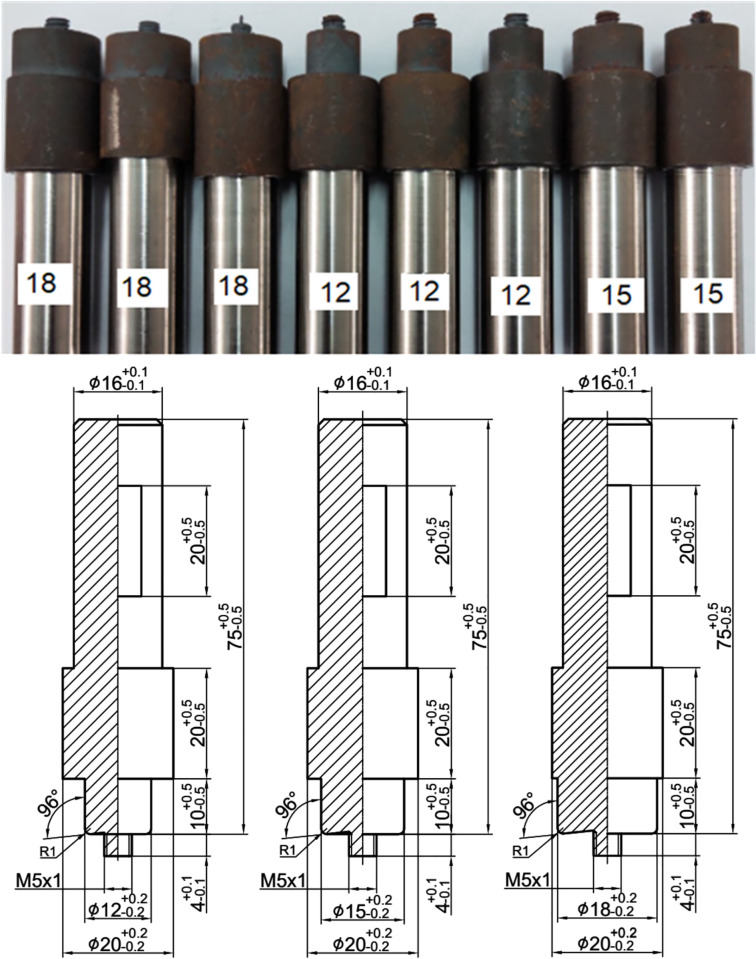
Welding tools with various shoulder diameters of 12, 15, 18 (mm).

Welding tools are made from H-13 tool steel material, which is the most widely used tool material [17,37]. As shown in Fig 3, the tool has a cylindrical stirring head (right thread M5x1) with shoulder diameters of 12.0, 15.0, and 18.0 mm, respectively.

### Heating equipment

**Fig 4:**
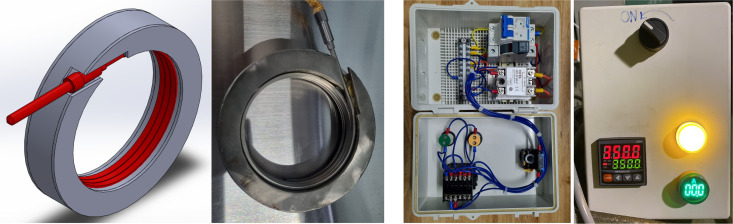
The resistance ring has thermal insulation and a heating control box.

The heating process before welding is to remove steam from the area to be welded. The heating temperature is usually just greater than the boiling temperature of water. Removing water from the joint helps eliminate porosity, brittleness, and cracking of the weld due to hydrogen produced during the decomposition of water at high temperatures when welding. Diminish the temperature differential between the welding heat source and the specimen. The heating process must ensure the heating of the entire welding area and the entire thickness of the welded object. Nowadays, heating devices have sensing, display, and temperature adjustment systems to help control weld heating and ensure welding quality. The heating device consists of two resistance rings with thermal insulation to ensure safety and a heating control box, as shown in Fig 4. The appropriate preheating temperature range for FSW of AA6061 aluminum alloys using resistance heating is typically between 100°C and 200°C [38–39]. This preheating helps reduce the welding forces required and enhances the weld quality by promoting better material flow and reducing thermal gradients that can lead to defects. This results in a more consistent and defect-free weld. Furthermore, preheating can minimize the risk of tool wear and increase the overall efficiency of the welding process. In this study, we heated and kept the workpiece stable at 100° C before friction stir welding. This temperature value is enough to remove water vapor from the welding area and increase weld quality for AA6061 material, consistent with previous studies [34,39].

### Welding jig

**Fig 5:**
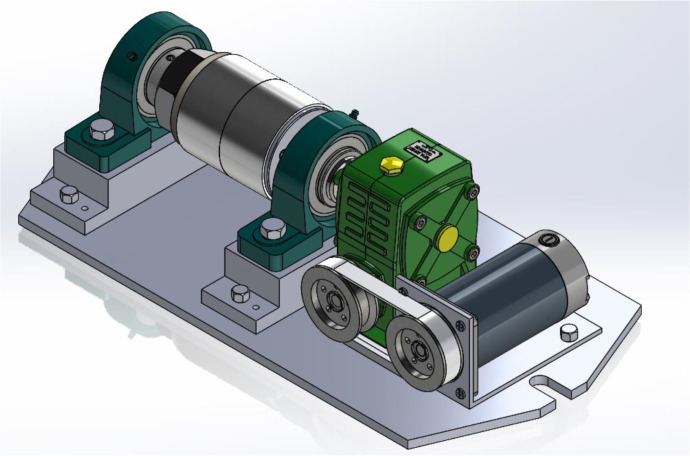
Fixture system.

The two aluminum pipe specimens are the same size and machined with a flat end surface. Two specimens are mounted on a mandrel designed to ensure rigidity and concentricity. Use the inside of the pipes to position them onto the mandrel, clamping the two specimens tightly with nuts. This mandrel is installed on the drive system with different speed levels, as shown in Fig 5. The core of the fixture comprises a robust mandrel, engineered to ensure both concentricity and rigidity of the specimens during the welding process. The tubular specimens are precisely positioned onto the mandrel using their inner diameters, and subsequently secured tightly by nuts, which exert axial clamping force. This mandrel assembly is then integrated into a drive system, enabling rotational control at various speed levels. The rotational motion is transmitted to the mandrel via a multi-stage power transmission system. Specifically, an electric motor provides the initial power, which is then transferred through a belt drive mechanism. This is followed by a worm gear drive, which precisely controls the rotational speed of the mandrel according to experimental requirements. Crucially, the fixture incorporates an in-situ preheating capability. Resistance heating coils are directly mounted onto the pipe specimens, allowing for localized preheating of the workpieces prior to the initiation of the FSW process. This preheating is intended to modify material properties and facilitate the welding operation. The entire apparatus, including the drive system, mandrel, and heating elements, is assembled and secured onto a base plate. This base plate is specifically designed for mounting onto the worktable of a milling machine, providing the necessary stability and precision for the FSW experiments. This integrated design allows for controlled rotation of the tubular workpieces while facilitating the application of preheating and the subsequent traverse of the FSW tool.

### Experimental procedure

**Fig 6:**
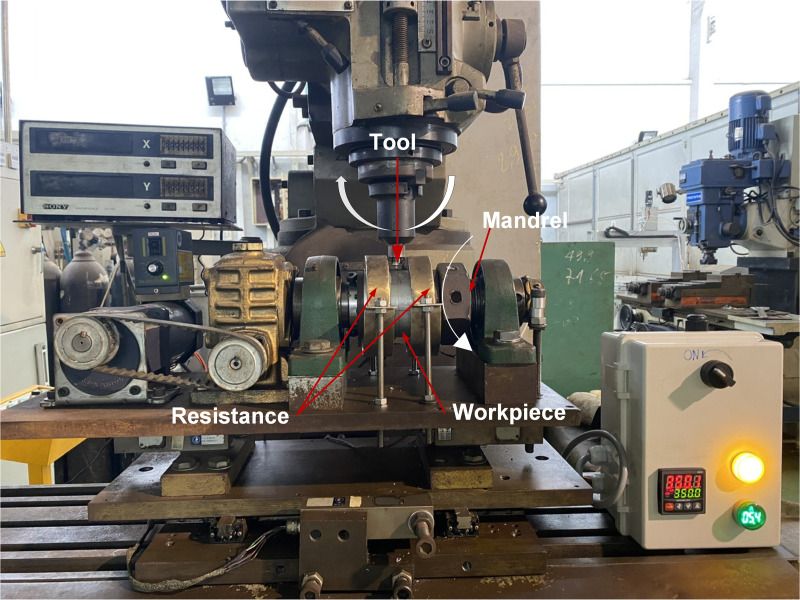
The RPFSW machine.

The basic principle of RPFSW in this study is described as follows: First, two aluminum alloy specimens are heated to 100 degrees Celsius by resistance. The resistance rings are shown in Fig 4, and the principle of RPFSW is shown in Fig 6. This heating method is similar to previous research [40]. As illustrated in Fig 3, the RPFSW procedure makes use of a non-consumable rotating tool with a specially created pin (probe) and shoulder. The tool’s design is crucial for effective material mixing and heat generation. The rotating tool is moved along the joint after being inserted into the joint line between two workpieces. Frictional heat produced by the rotating motion softens the material without melting it. The material surrounding the pin becomes plasticized (softened) as the tool travels along the joint due to the frictional heat. The shoulder applies a downward force, containing the softened material and preventing it from expelling. A pipe weld joint is formed after the tool moves along the welding direction. The RPFSW machine is shown in Fig 6.

The RPFSW technique differs from conventional FSW in that it uses heating as an additional resistance heat source to improve the thermoplasticization of the welding material, increase the quality of the weld joint, reduce defects, and prolong the life of the welding tool [28,41]. In order to accurately determine the impact of technological parameters on weld quality, it is necessary to experiment on actual models, thereby analyzing and evaluating the obtained data to ensure accuracy and reliability.

Conducting RPFSW experiment for AA6061 pipes, experimenting with three input parameters: tool rotation speed *n*, transverse speed *v*, and tool shoulder diameter *d*. A three-level, three-factor composite design involves evaluating the effects of three independent variables, each at three levels, on the response of the tensile strength (*y*) of the weldment. The main objective is to identify the optimal combination of these factors to maximize the tensile strength. The values of the input parameters are shown in Table 3.

**Table 3:** Technical parameters of the RPFSW process.

	Low	Center Point	High
Tool rotation speed n (rpm)	1250	1500	1750
Transverse speed v (mm/min)	75	87.5	100
Tool shoulder diameter d (mm)	12	15	18

Choose the optimal parameter y (tensile strength σ_k_), and three factors that affect the optimal parameter are rotation speed *n* (x_1_), transverse speed *v* (x_2_), and tool shoulder diameter *d*.


$$\left(x_{3}\right).\text{Where},\;\;x_{1}=\frac{n-1500}{250}\;;\;\;\;x_{2}=\frac{v-87.5}{12.5};\;\;x_{3}=\frac{d-15}{\text{3}}$$ (1)

Choose a full quadratic regression equation model for three factors (k = 3), and conduct full Box-Behnken experimental planning. The number of experiments to be performed is m = 15. Table 4 shows the experimental matrix following the Box-Behnken model with three factors.

**Table 4:** The experimental matrix.

Run order	Factors			Quadratic			Couple interaction		
	x_1_	x_2_	x_3_	x_1_^2^	x_2_^2^	x_3_^2^	x_1_x_2_	x_1_x_3_	x_2_x_3_
1	−1	−1	0	1	1	0	1	0	0
2	1	−1	0	1	1	0	−1	0	0
3	−1	1	0	1	1	0	−1	0	0
4	1	1	0	1	1	0	1	0	0
5	−1	0	−1	1	0	1	0	1	0
6	1	0	−1	1	0	1	0	−1	0
7	−1	0	1	1	0	1	0	−1	0
8	1	0	1	1	0	1	0	1	0
9	0	−1	−1	0	1	1	0	0	1
10	0	1	−1	0	1	1	0	0	−1
11	0	−1	1	0	1	1	0	0	−1
12	0	1	1	0	1	1	0	0	1
13	0	0	0	0	0	0	0	0	0
14	0	0	0	0	0	0	0	0	0
15	0	0	0	0	0	0	0	0	0

**Fig 7:**
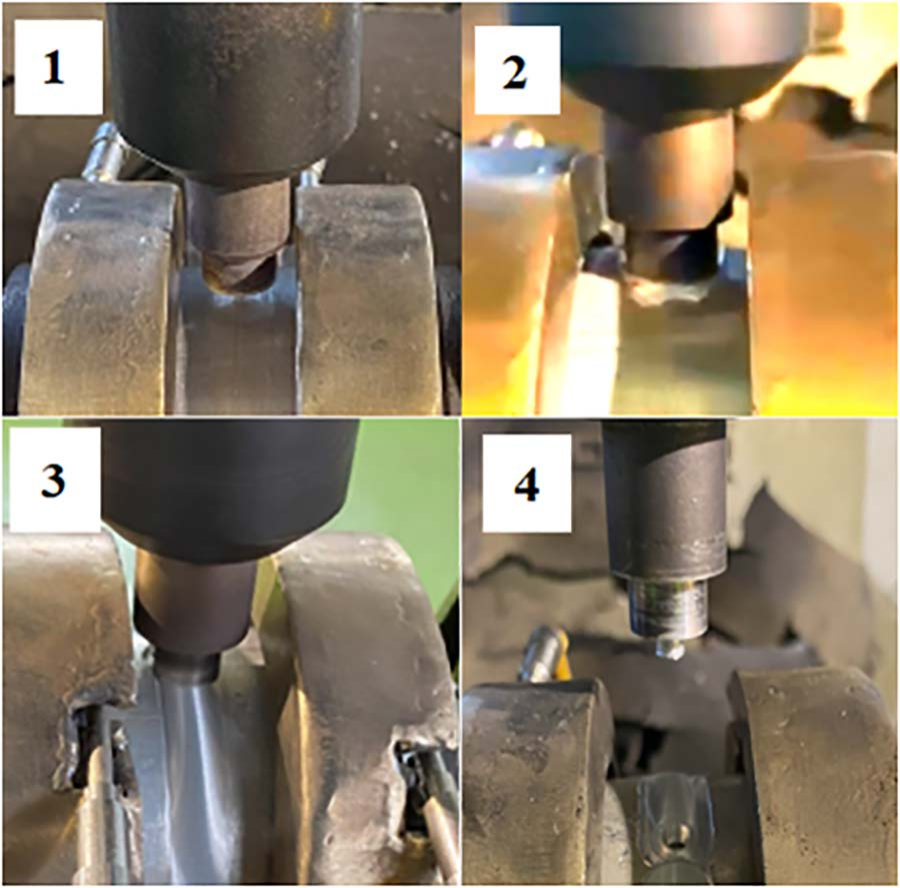
Weld formation process.

Fig 7 shows the heat-assisted friction stir welding process. This process is described as follows:

Step 1: The workpiece (after heating to 100°C) slowly moves up along the bed and comes into contact with the FSW tool, creating friction that generates heat at the contact surface. During the FSW process, the tool is inserted into the material to a depth corresponding to the pin length. The rotating tool generates frictional heat, which plasticizes the material without reaching its melting point. This softened material is then stirred and consolidated by the tool rotation and traverse movement along the joint line, resulting in a solid-state weld.

Step 2: When the temperature reaches the plastic state of the aluminum workpiece, start rotating the workpiece.

Step 3: The welding process is started until the aluminum tube rotates to complete a round.

**Fig 8:**
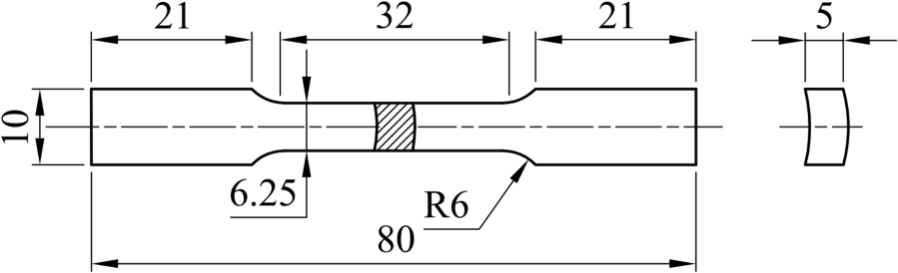
Geometries of tension specimens (dimensions in mm).

**Fig 9:**
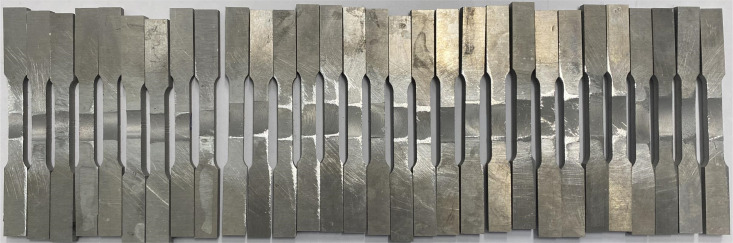
Tensile test samples.

Step 4: The FSW tool will stop at the end of the journey, and the bed will go down. After welding, the aluminum alloy pipe will be cut into eight parts; each cut part is processed on a wire electrical discharge machining to produce the sample for the tensile test, as shown in Fig 8, according to the international standard ISO 6892–1:2019 [42]. The machined samples for the tensile test are shown in Fig 9.

**Fig 10:**
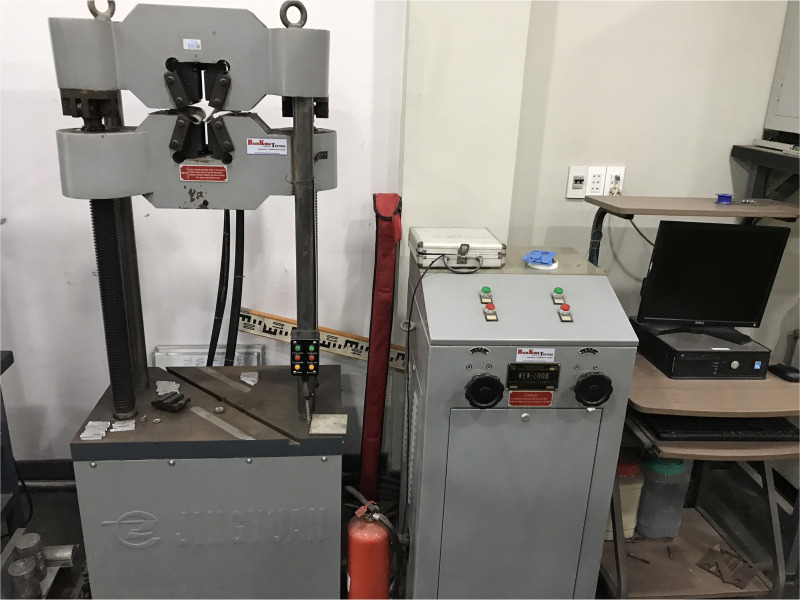
Tensile testing machine.

The tensile testing process is conducted on a Jingyuan universal testing machine (WEW-100B model), as shown in Fig 10. This tensile test machine is used in materials laboratories and tested with a maximum force of 100 kN.

The experimental matrix for the three factors (n, v, d) in the encoded form of the parameters was created using Minitab software [43]. There are 15 experiments, as shown in Table 4. There are three tensile samples tested for each experiment. So, there are 45 actual tensile samples.

## Results and discussion

**Fig 11:**
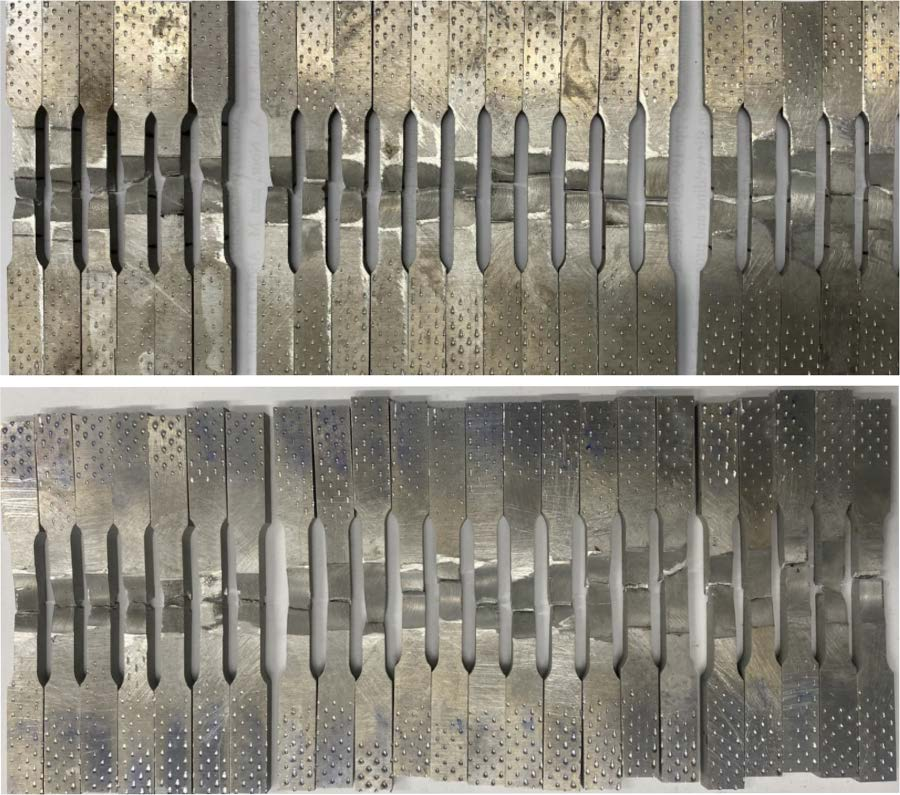
Samples after the tensile test.

After the tensile test, the samples had the shape as shown in Fig 11.

The results of measuring the tensile strength value of 15 experiments, each experiment performed 03 measurements, and the average value of the measurements is exhibited in Table 5.

**Table 5:** The experimental matrix for the three factors and their corresponding responses.

Run order	Factors			Average value
	n (rpm)	v (mm/min)	d (mm)	$\overset{―}{y_{i}}$
1	1250	75	15	148.100
2	1750	75	15	174.200
3	1250	100	15	163.867
4	1750	100	15	195.200
5	1250	87.5	12	136.233
6	1750	87.5	12	167.933
7	1250	87.5	18	157.033
8	1750	87.5	18	152.867
9	1500	75	12	164.300
10	1500	100	12	188.467
11	1500	75	18	185.200
12	1500	100	18	175.900
13	1500	87.5	15	177.400
14	1500	87.5	15	179.400
15	1500	87.5	15	178.400

From the results in Table 5, using Minitab software to process the data, the regression model analysis results are revealed in Table 6.

**Table 6:** Results of regression model analysis.

Term	Effect	Coef	SE Coef	T-Value	P-Value	VIF
Constants		178.40	3.41	52.30	0.000	
x_1_	21.24	10.62	2.09	5.08	0.004	1.00
x_2_	12.91	6.45	2.09	3.09	0.027	1.00
x_3_	3.52	1.76	2.09	0.84	0.438	1.00
x_1_*x_1_	−33.01	−16.50	3.07	−5.37	0.003	1.01
x_2_*x_2_	16.89	8.45	3.07	2.75	0.040	1.01
x_3_*x_3_	−16.76	−8.38	3.07	−2.73	0.042	1.01
x_1_*x_2_	2.62	1.31	2.95	0.44	0.676	1.00
x_1_*x_3_	−17.93	−8.97	2.95	−3.04	0.029	1.00
x_2_*x_3_	−16.73	−8.37	2.95	−2.83	0.037	1.00

Based on the regression coefficient value provided by the software, the column (Coef) in Table 6 gives the constant term and the coefficient of the input parameters a regression equation relating the three welding technology parameters *n, v, d,* and *y* (tensile stress σ_k_).

The quadratic regression equation:


$$\begin{array}{c} y=178.4+10.62x_{1}+6.45x_{2}+1.76x_{3}-16.5x_{1}^{2}+\\ +8.45x_{2}^{2}-8.38x_{3}^{2}+1.31x_{1}x_{2}-8.97x_{1}x_{3}-8.37x_{2}x_{3} \end{array}$$ (2)

_After removing insignificant parameters (P > 0.05), the regression equation in uncoded is as follows:_


$$y=-891+1.014n-5.6v+66d-0.000264n^{2}+0,0541v^{2}-0.931d^{2}-0.01196nd-0.2231vd$$ (3)

**Fig 12:**
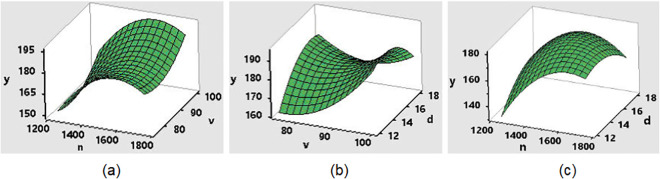
Effect of technological parameters on tensile strength. (a) Effect of *n* and *v*; (b) Effect of *v* and *d*; (c) Effect of *n* and *d*.

Fig 12 shows the relationship between *n*, *v*, and *d* to weldment tensile strength (*y*). Fig 12(a) shows the relationship between *n* and *v* on the tensile strength *y*. As *n* increases, *y* also increases and reaches a certain tensile limit, then stabilizes and slightly decreases as *n* continues to increase. Meanwhile, *y* increases as *v* increases. The maximum values of the tensile strength *y* are found when *n* reaches a value of about 1628 rpm and *v* reaches a value of 100 mm/min, indicating that there is an optimal interaction between these two parameters to maximize tensile. The tensile strength of the weld depends on the welding conditions, specifically on the temperature and material flow caused by the friction stir weld. Indeed, we also investigated the effect of rotation speed n with many different values. When *n* is less than 1050 rpm, the temperature generated by friction stirring is small, and the tool does not generate enough heat to form a weld, as shown in S1 Fig. When increasing the number of rotation speeds *n*, the temperature of the welding area increases, leading to improved weld quality. When *n* increased to 2500 rpm, the weld joint was overheated, leading to poor weld quality, as shown in S2 Fig. Therefore, rotation speed significantly affects the heat generated by friction stirring and the weld’s quality.

**Fig 13:**
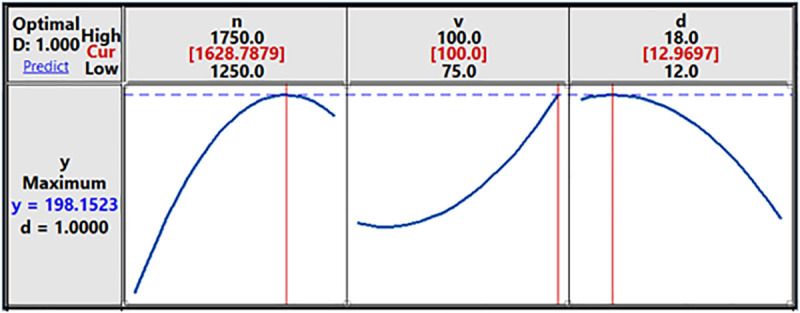
Graph determining the optimal value of each technological parameter.

Fig 12(b) presents the effect of *v* and *d* on *y*. An optimal combination of transverse speed and shoulder diameter ensures adequate heat generation, material flow, and consolidation. This balance is critical for achieving high tensile strength. The results show that a rise in *v* leads to a rise in *y*, but is less evident with decreasing *d* values. Fig 12(c) shows the effect of *n* and *d* on tensile strength. Similar to the graph in Fig 12a, the increase in *n* correlates with the increase in *y* to the greatest strength; if *n* continues to increase, the *y* value will decrease. The rotation speed impacts the material flow, heat generation, and ultimate strength of the welded joint. Increasing rotation speed up to an optimal point generally enhances tensile strength, beyond which it may decline due to overheating and defect formation. For shoulder diameter, when *d* increases from 12 to 13, *y* increases, but when *d* increases beyond the value 13, *y* decreases. This demonstrates that the tool’s shoulder diameter has an impact on weld strength as well. The shoulder diameter affects the pressure applied on the workpiece and the forging action that consolidates the material. Adequate pressure is crucial for eliminating voids and achieving a defect-free weld. Minitab’s Response Optimizer tool was used to find optimal working parameters. The graph determining the optimal value of each technological parameter with the above objective function is shown in Fig 13.

The results show that the friction stir weld of the AA6061 aluminum alloy cylinder was successfully fabricated, and the tensile strength relationship was determined under experimental conditions of welding technology parameters (*n*, *v*, *d*), with weldment tensile strength reaching from 49.7% to 72.4% compared to the base material. It points out that the tensile strength of the welded joint reaches the highest value of σ_k_ = 198.15 MPa corresponding to the processing parameters of rotation speed 1629 rpm, transverse speed 100 mm/min, and tool shoulder diameter 13 mm.

For an overview of heat-assisted friction stir welded joints durability studies, Table 7 compares the results of this study with previous studies with the same material AA6061. Table 7 shows that our results are quite consistent with previous studies. Furthermore, the durability of our welded joints is higher than that of previous studies. We hope our results are informative and can be used for further research on preheated friction stir welding of AA6061 material.

**Table 7:** Comparison with other findings.

Materials	n (rpm)	v (mm/min)	d (mm)	Temperature (°C)	Ultimate stress (MPa)	Ref.
AA 6061	1120	30	24	100	114	Omer et al. [39]
AA 6061	1400	30	24	100	93	Omer et al. [39]
AA 6061	1800	30	24	100	80	Omer et al. [39]
AA 6061	500	125	12.5	160	134	Kalinenko et al. [34]
AA 6061	750	125	12.5	160	137	Kalinenko et al. [34]
AA 6061	1100	125	12.5	160	138	Kalinenko et al. [34]
AA 6061	1250	75	15	100	148.1	This study
AA 6061	1250	87.5	12	100	136.2	This study
AA 6061	1500	87.5	15	100	178.4	This study
AA 6061	1750	100	15	100	192.5	This study

**Fig 14:**
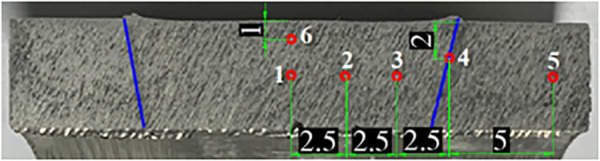
Joint cross-section at reasonable welding conditions and microstructure inspection positions (mm).

With the above results, it is necessary to check the microscopic structure in the weld center area to evaluate the best weld quality. A weld sample under a parameter process of n = 1500 rpm, v = 100 mm/min, d = 12 mm is used as a representative to examine the microstructural changes in the RFSW weld. The microstructure of the weld is checked at points 1, 2, 3, 4, 5, 6, as shown in Fig 14. The microscopic examination is performed on a Buehler ViewMet optical microscope.

**Fig 15:**
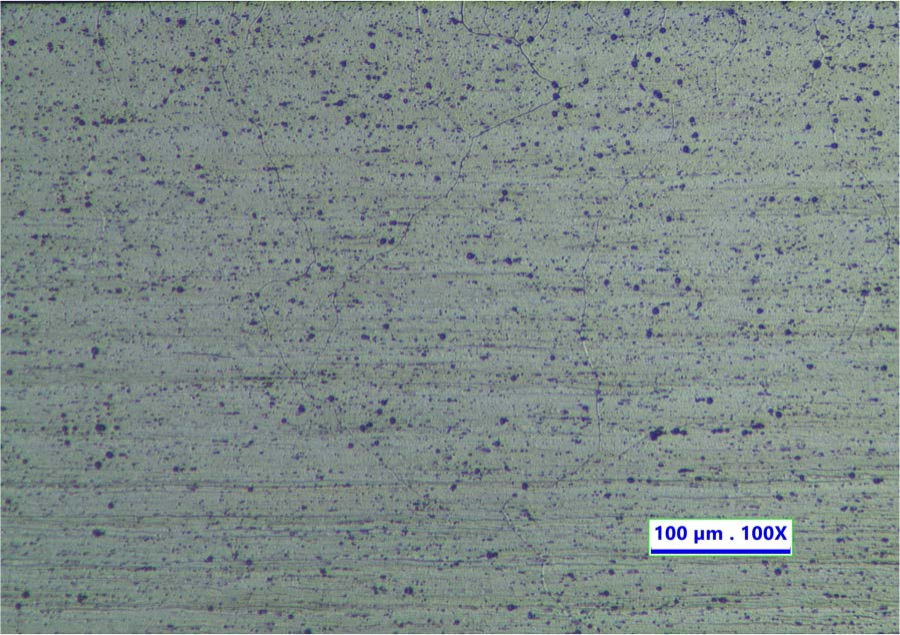
Microstructure of parent material.

The results of the microscopic structural examination were photographed using an optical microscope with magnification up to 1000 times. The initial metal workpiece is called parent material (PM). The microstructure of PM has a coarse and long grain structure, as exhibited in Fig 15.

**Fig 16:**
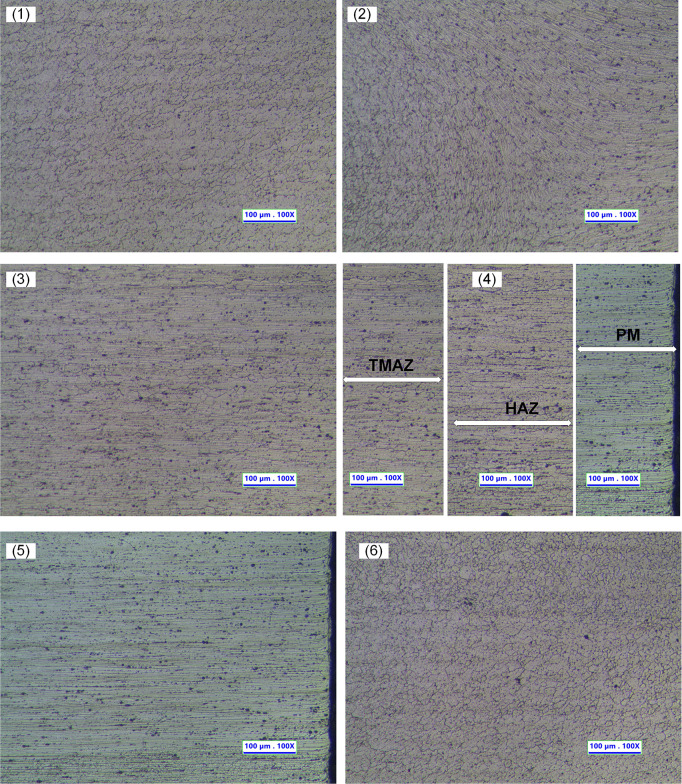
Microstructure at different positions in the weld zone: weld center area (1,2,6); thermomechanical affected area (3); heat affected zone (4); base metal region (5).

Fig 16 illustrates the microstructure at different positions in the weld zone: weld center area (1,2,6), thermomechanical affected area (3), heat affected zone (4), and base metal region (5). Weld center region (WN – Weld Nugget): microstructure at position 1 (similar to microstructure at position 6) is the point located at the center line of the weld due to intense plastic deformation by impact by using the stirring tool. This area has a fine-grained structure in the direction from the weld root to the weld surface, demonstrating increased tensile strength during the RFSW process. The microstructure at position 2 has a fine grain size similar to the weld center area (left), and the right side is similar to the thermo-mechanically affected zone. The microstructure at position 3 indicates the thermo-mechanically affected zone (TMAZ). It lies between the heat-affected zone (HAZ) and the weld nugget. The material in the TMAZ is subjected to elevated temperatures due to the heat generated during the process. In the thermo-mechanically affected zone, deformation occurs due to frictional heat and the mechanical impact of the stirring tool. However, the temperature is not high enough to cause complete recrystallization. In addition to exposure to heat, TMAZ also undergoes significant plastic deformation due to mechanical forces applied during this process. This leads to the particles in TMAZ often being elongated in a certain direction depending on the direction of rotation of the stirrer, consistent with previous studies [35].

The microstructure at position 4 indicates the heat-affected zone (HAZ). The HAZ is located adjacent to the weld metal or the melted zone. It extends from the fusion line, where the base material has melted and solidified, to the unaffected base material. The material in the HAZ experiences a range of temperatures. The temperature gradient decreases from the fusion line to the base material. These temperatures are high enough to alter the microstructure but not to melt the material. Higher temperatures have the potential to induce grain development, producing coarser grains than the base material.

The microstructure at position 5 indicates the unaffected zone (UZ). The material’s microstructure and mechanical properties remain unchanged since it is situated distant from the weld, is not distorted, and is not impacted by heat.

It can be concluded that with the above optimal friction stir welding technology parameters, the grain structure change process is quite clear and intense between different regions. This certainly increases the mechanical characteristics of friction stir welds.

**Fig 17:**
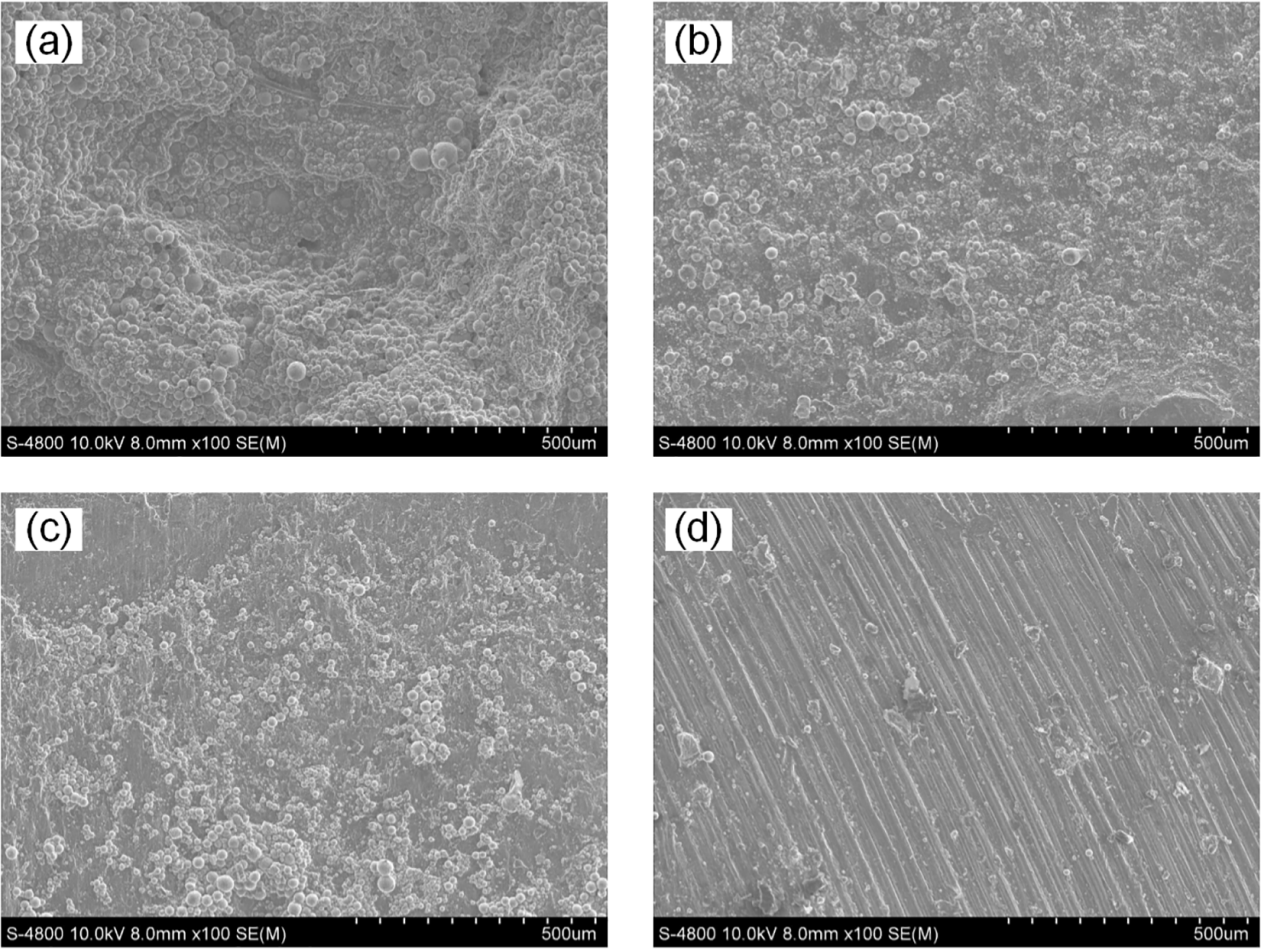
SEM image of the fracture surface of (a) material AA6061 (unwelded); (b) the joint with n = 1500, v = 100, d = 12; (c) the joint with n = 1500, v = 87.5, d = 15; (d) the joint with n = 1500, v = 100, d = 18.

Fig 17(a) presents the SEM image of the fracture surface of material AA6061 (unwelded). The fracture surface of the unwelded AA6061 material shows a rough and irregular morphology, indicating a ductile fracture mode. The presence of dimples and voids suggests plastic deformation before fracture, typical of ductile failure. The grains appear to be relatively fine, which is consistent with a material that has not undergone extensive thermal processing. Fig 17(b) shows the SEM image of the fracture surface of the joint with n = 1500 rpm, v = 100 mm/min, d = 12 mm. The fracture surface appears more uniform compared with other joints, with predominantly ductile features, such as numerous small dimples. Grain refinement is evident, likely due to optimal heat input and material flow during welding, leading to a homogenous microstructure. Fig 17(c) presents the SEM image of the fracture surface of the joint with n = 1500 rpm, v = 87.5 mm/min, d = 15 mm. The fracture surface exhibits a combination of ductile and brittle characteristics. Some areas with dimples indicate ductile failure, but also smoother regions indicate brittle fracture. The transition between ductile and brittle regions suggests variations in temperature and stress during the welding process. Fig 17(d) shows the SEM image of the fracture surface of the joint with n = 1500 rpm, v = 100 mm/min, d = 18 mm. The fracture surface shows elongated features aligned in a specific direction, suggesting a mixed mode of fracture with predominant brittle features. The elongated and striated appearance indicates a combination of plastic deformation and some brittle failure, potentially due to localized overheating or improper material flow. The result shows that the welding conditions significantly influence the fracture surface morphology and grain structure. Changing the transverse speed and tool shoulder diameter alters the heat input and material flow, affecting the resultant microstructure and mechanical properties. Optimal welding conditions lead to a more homogeneous and ductile microstructure, enhancing the tensile strength and overall performance of the weld. Conversely, non-optimal conditions can result in inhomogeneous grain structures and mixed fracture modes, compromising the mechanical characteristics of the joint [44,45].

## Conclusions

This study investigated resistance preheated friction stir welding of AA6061 aluminum alloy pipes with process parameters optimized using a Box–Behnken design. The results showed that joint tensile strength strongly depends on traverse speed, tool rotation speed, and shoulder diameter. The optimum condition—rotation speed of 1629 rpm, traverse speed of 100 mm/min, and shoulder diameter of 13 mm—yielded a maximum tensile strength of 198.15 MPa (≈72% of the base material strength). The microstructure of the material depends on the position of the weldment. In the center of the weld (weld nugget), the grain size is fine. The particles in TMAZ are often elongated in a certain direction depending on the tool’s rotation direction. The grains in the HAZ are coarser compared to the base material. Overall, the findings demonstrate that RPFSW, with appropriate parameter selection, can significantly enhance joint strength and quality in AA6061 aluminum pipes, offering a promising alternative to conventional joining methods.

## Supporting information

S1 Fig The weld joint does not have enough heat when stirring at a low speed. (TIF)

S2 Fig The weld joint was overheated when stirring at high speed. (TIF)

S3 Dataset. (DOCX)
