# Supplementary figures and images for: Effect of technological parameters on mechanical properties and microstructure of heat-assisted friction stir welded joints of 6061 aluminum alloy
Source: PLoS One. 2025 Oct 22;20(10):e0334979. doi: 10.1371/journal.pone.0334979 (PMC12543162; doi:10.1371/journal.pone.0334979)

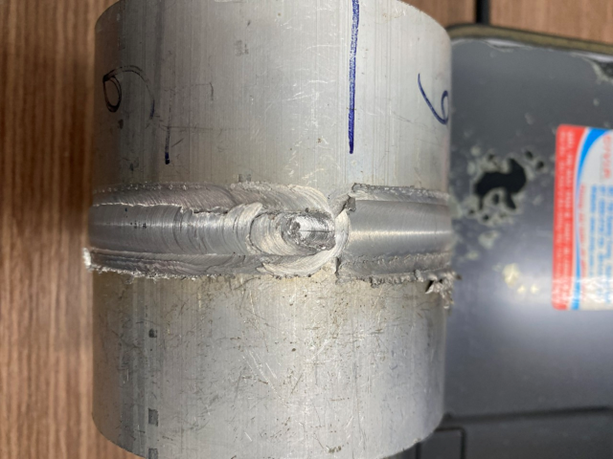

Supplement: S1 Fig — (TIF) [file pone.0334979.s001.tif]

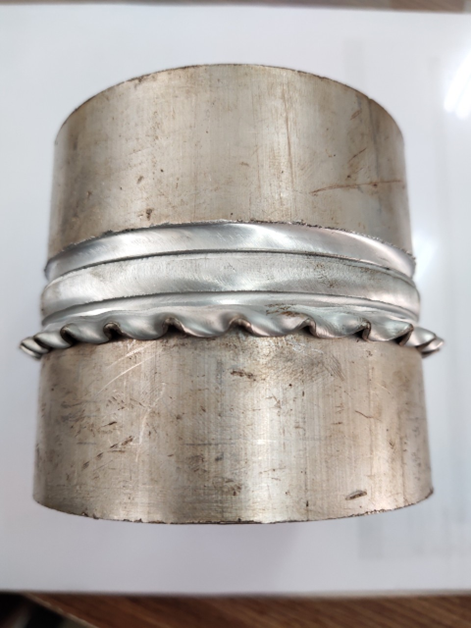

Supplement: S2 Fig — (TIF) [file pone.0334979.s002.tif]
