# Supplementary material for: Effect of technological parameters on mechanical properties and microstructure of heat-assisted friction stir welded joints of 6061 aluminum alloy
Source: PLoS One. 2025 Oct 22;20(10):e0334979. doi: 10.1371/journal.pone.0334979 (PMC12543162; doi:10.1371/journal.pone.0334979)
Supplement: S3 — (DOCX) [file pone.0334979.s003.docx]

**Table 1**. Chemical composition of AA6061 material.

| Element | Al | Zn | Mg | Cu | Si | Fe | Mn | Cr | Ti | Others |
| --- | --- | --- | --- | --- | --- | --- | --- | --- | --- | --- |
| Composition (%) | 97.3 | 0.32 | 0.71 | 0.16 | 0.6 | 0.48 | 0.23 | 0.04 | 0.02 | 0.14 |

**Table 2**. Mechanical properties of AA6061 material

| Yield strength (MPa) | Ultimate strength (MPa) | Elongation (%) |
| --- | --- | --- |
| 230.2 | 273.8 | 12 |

**Table 3**. Technical parameters of the RPFSW process

|  | Low | Center Point | High |
| --- | --- | --- | --- |
| Tool rotation speed n (rpm) | 1250 | 1500 | 1750 |
| Transverse speed v (mm/min) | 75 | 87,5 | 100 |
| Tool shoulder diameter d (mm) | 12 | 15 | 18 |

**Table 4**. The experimental matrix

| **Run order** | **Factors** | | | **Quadratic** | | | **Couple interaction** | | |
| --- | --- | --- | --- | --- | --- | --- | --- | --- | --- |
|  | **x_1_** | **x_2_** | **x_3_** | **x_1_^2^** | **x_2_^2^** | **x_3_^2^** | **x_1_x_2_** | **x_1_x_3_** | **x_2_x_3_** |
| 1 | -1 | -1 | 0 | 1 | 1 | 0 | 1 | 0 | 0 |
| 2 | 1 | -1 | 0 | 1 | 1 | 0 | -1 | 0 | 0 |
| 3 | -1 | 1 | 0 | 1 | 1 | 0 | -1 | 0 | 0 |
| 4 | 1 | 1 | 0 | 1 | 1 | 0 | 1 | 0 | 0 |
| 5 | -1 | 0 | -1 | 1 | 0 | 1 | 0 | 1 | 0 |
| 6 | 1 | 0 | -1 | 1 | 0 | 1 | 0 | -1 | 0 |
| 7 | -1 | 0 | 1 | 1 | 0 | 1 | 0 | -1 | 0 |
| 8 | 1 | 0 | 1 | 1 | 0 | 1 | 0 | 1 | 0 |
| 9 | 0 | -1 | -1 | 0 | 1 | 1 | 0 | 0 | 1 |
| 10 | 0 | 1 | -1 | 0 | 1 | 1 | 0 | 0 | -1 |
| 11 | 0 | -1 | 1 | 0 | 1 | 1 | 0 | 0 | -1 |
| 12 | 0 | 1 | 1 | 0 | 1 | 1 | 0 | 0 | 1 |
| 13 | 0 | 0 | 0 | 0 | 0 | 0 | 0 | 0 | 0 |
| 14 | 0 | 0 | 0 | 0 | 0 | 0 | 0 | 0 | 0 |
| 15 | 0 | 0 | 0 | 0 | 0 | 0 | 0 | 0 | 0 |

**Table 5**. The experimental matrix for the three factors and their corresponding responses

| **Run order** | **Factors** | | | **Average value** |
| --- | --- | --- | --- | --- |
|  | **n** (rpm) | **v** (mm/min) | **d** (mm) | $\bar{\boldsymbol{y}_{\boldsymbol{i}}}$ |
| 1 | 1250 | 75 | 15 | 148.100 |
| 2 | 1750 | 75 | 15 | 174.200 |
| 3 | 1250 | 100 | 15 | 163.867 |
| 4 | 1750 | 100 | 15 | 195.200 |
| 5 | 1250 | 87.5 | 12 | 136.233 |
| 6 | 1750 | 87.5 | 12 | 167.933 |
| 7 | 1250 | 87.5 | 18 | 157.033 |
| 8 | 1750 | 87.5 | 18 | 152.867 |
| 9 | 1500 | 75 | 12 | 164.300 |
| 10 | 1500 | 100 | 12 | 188.467 |
| 11 | 1500 | 75 | 18 | 185.200 |
| 12 | 1500 | 100 | 18 | 175.900 |
| 13 | 1500 | 87.5 | 15 | 177.400 |
| 14 | 1500 | 87.5 | 15 | 179.400 |
| 15 | 1500 | 87.5 | 15 | 178.400 |

**Table 6.** Results of regression model analysis

| Term | Effect | Coef | SE Coef | T-Value | P-Value | VIF |
| --- | --- | --- | --- | --- | --- | --- |
| Constants |  | 178.40 | 3.41 | 52.30 | 0.000 |  |
| x_1_ | 21.24 | 10.62 | 2.09 | 5.08 | 0.004 | 1.00 |
| x_2_ | 12.91 | 6.45 | 2.09 | 3.09 | 0.027 | 1.00 |
| x_3_ | 3.52 | 1.76 | 2.09 | 0.84 | 0.438 | 1.00 |
| x_1_*x_1_ | -33.01 | -16.50 | 3.07 | -5.37 | 0.003 | 1.01 |
| x_2_*x_2_ | 16.89 | 8.45 | 3.07 | 2.75 | 0.040 | 1.01 |
| x_3_*x_3_ | -16.76 | -8.38 | 3.07 | -2.73 | 0.042 | 1.01 |
| x_1_*x_2_ | 2.62 | 1.31 | 2.95 | 0.44 | 0.676 | 1.00 |
| x_1_*x_3_ | -17.93 | -8.97 | 2.95 | -3.04 | 0.029 | 1.00 |
| x_2_*x_3_ | -16.73 | -8.37 | 2.95 | -2.83 | 0.037 | 1.00 |
